# Food Adulteration

**Published:** 1859-08

**Authors:** 


### FOOD ADULTERATION.

Much has been written on this subject of late, with less of fact than fiction. Some adulterations are positively beneficial, and the greater the adulteration the greater the benefit.—When a druggist puts twenty-five per cent. of flour, which costs three cents a pound, into his morphia, which sells at sixteen dollars a pound, not only he is benefitted by the adulteration, but the person who swallows it; on the principle, that the less medicine a man takes the better.

When a milkman puts one-eighth the nominal per centage of the pure Croton in each gallon of his swill stuff, who does not see that both seller and purchaser are benefitted.

When the distiller adulterates his liquors with strychnine, and coculus indicus, and arsenic, with other poisons too tedious to mention, he is benefitted pecuniarily; society morally; and the drinker's friends and family socially; for a drunkard is of no account, and the sooner he is killed off, the fewer sins he will have to answer for, and the sooner will society have a happy deliverance of the burden.

The art of adulterating food is daily becoming more and more of a science in London; and yet in London the average duration of human life is steadily increasing. These are the simple facts of the case, and we account for them in the goodness and wisdom of that Power which made the world; and destined man, his child, to live upon, and occupy, and conquer it. The human body is so contrived, that it can wonderfully adapt itself to the conditions under which it is placed, and becomes in time capable of rendering itself invulnerable by noxious agencies. A large proportion of all the medicines used lose their power, and become inert, by continued exhibition. Hence the expression employed millions of times, "it seemed to do me good at first."

On the other hand, adulterations of food are most generally made with harmless articles, or those which are coarser. Milk is not made injurious as food by having water mixed with it, nor coffee with having chickory.

Therefore we invite our readers not to be particularly alarmed by statements made in the papers on the subject of the adulteration of food, more with a view of raising a sensation and "the wind," at the same time. A great parade of learning and of scientific research is sometimes made with very little profit, yet as hurtful ingredients are sometimes mixed with our food and our drinks, we counsel those who are fond of being always on the safe side to bake their own bread and brew their own beer, and then they will have the satisfaction of knowing that they are eating bread made of flour instead of alum and saleratus; and drinking wine made of the pure juice of the grape, instead of boiled logwood.

A still greater advance would be made in promoting the general health of the people, by the simplification of dishes. Let every dish be composed of a single article of food; if it be of potatoes, let it be of potatoes and nothing else; let rice be rice, and soup soup; let every meat be fresh; let every vegetable be perfect, ripe, recent; let every thing of the fish kind, especially in summer, be seen "a kicking" within the halt hour of cooking; and let three articles of food, at any one meal, be the largest allowable variety; for it is variety of food at each meal, which is the great tempter to excess in quantity; the great founder of that dyspepsia which is the torment, greater or less, of half the people of any civilized nation.

It will be a difficult matter for any person in ordinary good health to eat too much at a single meal made of two or three articles of food. It is worth trying.

